# Racial disparities in patient survival and tumor mutation burden, and the association between tumor mutation burden and cancer incidence rate

**DOI:** 10.1038/s41598-017-13091-y

**Published:** 2017-10-20

**Authors:** Wensheng Zhang, Andrea Edwards, Erik K. Flemington, Kun Zhang

**Affiliations:** 1 Department of Computer Science, Bioinformatics facility of Xavier RCMI Center of Cancer Research, Xavier University of Louisiana, 1 Drexel Drive, New Orleans, LA 70125 USA; 2 Tulane School of Medicine, Tulane Cancer Center, Tulane University, 1700 Tulane Ave, New Orleans, LA 70112 USA

## Abstract

The causes underlying racial disparities in cancer are multifactorial. In addition to socioeconomic issues, biological factors may contribute to these inequities, especially in disease incidence and patient survival. To date, there have been few studies that relate the disparities in these aspects to genetic aberrations. In this work, we studied the impacts of race on the patient survival and tumor mutation burden using the data released by the Cancer Genome Atlas (TCGA). The potential relationship between mutation burden and disease incidence is further inferred by an integrative analysis of TCGA data and the data from the Surveillance, Epidemiology, and End Results (SEER) Program. The results show that disparities are present (p < 0.05) in patient survival of five cancers, such as head and neck squamous cell carcinoma. The numbers of tumor driver mutations are differentiated (p < 0.05) over the racial groups in five cancers, such as lung adenocarcinoma. By treating a specific cancer type and a racial group as an “experimental unit”, driver mutation numbers demonstrate a significant (*r* = 0.46, *p* < 0.002) positive correlation with cancer incidence rates, especially when the five cancers with mutational disparities are exclusively focused (*r* = 0.88, *p* < 0.00002). These results enrich our understanding of racial disparities in cancer and carcinogenic process.

## Introduction

Eliminating racial disparities in cancer screening, diagnosis, treatment and mortality is an essential step toward the improvement of health outcomes for all cancer patients in America^1^. The promise of this objective depends on identifying and addressing the multifactorial reasons underlying the disparities. It is well recognized that socioeconomic issues, such as income and treatment delays, play a critical role in the high mortality of several cancers in minority populations^2^. Meanwhile, some studies show that biological factors may contribute to these inequities, especially in disease incidence and patient survival^3^.

Previous studied have associated the race-related survival stratification of cancer patients to the differences of genetic alterations present in tumor cells. Carethers *et al*.^4^ showed that the frequency of microsatellite instability (MSI) among African American colon cancers is half of that of MSI for the Caucasian counterpart. The authors proposed that, because MSI is associated with good survival for colon cancer patients, the relative lack of MSI in African American patients could be related to the high morality. Keenan *et al*.^5^ reported that racial differences in TP53 mutation, PAM50 basal subtype and triple-negative tumor prevalence influence the magnitude and significance of racial disparity in tumor recurrence of breast cancer. Petrovics *et al*.^6^ observed distinct prevalence between African American (AA) and Caucasian American prostate cancer (CaP) genomes in three recurrent genomic alterations, which occurred in the genes (loci) PTEN, LSAMP region and ERG. They further found that a novel deletion of the LSAMP locus, as a prevalent genomic alteration in AA CaP, was associated with rapid disease progression.

In this study, we first used the data released by the Cancer Genome Atlas (TCGA) to estimate the effect of race on patient survival time and mutation burden of tumors in 16 cancer types (subtypes). Then, we extended the analysis to the determination of potential relationship between mutation burden and disease incidence, a less investigated issue, by integrating TCGA data and the data from the Surveillance, Epidemiology, and End Results (SEER) Program. The results obtained from this study enrich our knowledge in cancer disparities and the related carcinogenic process.

## Material and Methods

### TCGA data

We downloaded the clinical and somatic data from the TCGA database (http://cancergenome.nih.gov/) on April 24, 2015. Those data, contributed by different institutes, are generated using various sequencing platforms, somatic mutation calling algorithms and computational tools. Except for ovarian carcinomas (OV), we choose one representative dataset for each cancer type according to the following criteria. First, the selected dataset contains the largest number of tumor samples (or patients). Second, if two or more datasets are of the same size, we choose the one in which the mutations are measured by the IlluminaGA DNASeq platform and are called by the latest automated system. Lastly, if the decision cannot be reached by the previous two steps, we select the dataset provided by the UCSC Genome Browser. For OV, we employ the datasets from Massachusetts Institute of Technology and Washington University in St. Louis. The basic information of the used somatic and clinical datasets is summarized in Supplementary Table 1. Synonymous mutations and those under the categories of “intron” and “rna” are excluded from further analysis.

### SEER data

Age-adjusted race-specific cancer incidence rates, based on the registries in 18 areas from 2008–2012 (or from 1992–2007 for glioblastoma multiforme (GBM)), are retrieved from the SEER website (http://seer.cancer.gov/). In the SEER review reports, cancers are categorized by tissue sites. For a TCGA cancer, if it is the absolutely-predominant subtype of a SEER cancer, the incidence rate (the number of new cancer cases per 100,000 individuals per year) in a racial group is estimated by the incidence rate of the SEER cancer. Otherwise, a race-specific incidence rate of the TCGA cancer (Cancer-A) is estimated by multiplying the incidence rate of the SEER cancer (Cancer-B) that covers Cancer-A with a weight that represents the proportion of the tumor cases of Cancer-A among the total cases of Cancer-B. When the SEER reports do not include the distribution of histological subtypes for a cancer, the weight information for estimating the incidence rates of a TCGA cancer is obtained from other literature. In particular, the data in Olshan *et al*.^7^ are used in estimating the incidence rates of KIRC and KIPC, and the data in Wright *et al*.^8^ and Dubrow & Darefsky^9^ are applied to the estimations for UCEC and GBM, respectively. The details regarding the adaptation of incidence rates from the SEER cancers to the TCGA cancers are described in Supplementary Table 2.

### Data of stem cell divisions

The lifetime number of stem cell divisions for eight cancer tissues (out of the 16 TCGA cancers summarized in Table 1) are estimated by Tomasetti and Vogelstein^10^. We directly use their estimations in this study.

**Table 1:** The summary of sample profiles^‡^.

Cancer	Total samples	White	Black	Asian
BLCA	382 (233)	300 (183)	22 (13)	42 (26)
GBM	594 (285)	505 (256)	50 (17)	13 (5)
HNSC	522 (504)	447 (439)	45 (36)	11 (11)
KIRC	533 (417)	466 (390)	51 (14)	8 (7)
LUAD	521 (488)	391 (385)	52 (29)	8 (8)
LUSC	496 (178)	341 (111)	31 (9)	9 (5)
BRCA	1080 (967)	747 (698)	172 (116)	61 (57)
OV	588 (371)	498 (324)	34 (17)	20 (12)
UCEC	538 (248)	372 (193)	104 (25)	20 (13)
COAD	455 (216)	214 (177)	54 (19)	11 (7)
THCA	506 (402)	329 (263)	27 (18)	52 (39)
CESC	305 (198)	210 (142)	30 (16)	19 (19)
ESCA	174 (171)	110 (109)	2 (2)	41 (41)
KIRP	272 (168)	189 (108)	60 (43)	5 (2)
LIHC	363 (197)	175 (120)	17 (14)	159 (54)
STAD	453 (288)	288 (167)	12 (4)	89 (76)

^‡^Outside the brackets are the numbers of samples with clinical information only. Inside the brackets are the numbers of samples with both clinical and genomic information. Some samples do not belong to any racial group of White, Black or Asian.

### Racial groups

The TCGA patients (or tumors) are partitioned into three racial groups, “White”, “Black” and “Asian”. We exclude the patients that do not belong to these groups. These groups are aligned to the SEER populations “White”, “Black” and “Asian and Pacific Islands”, respectively.

### Statistical analysis

We use R to perform all statistical analyses. The race-specific Kaplan-Meier survival curves are created by the function “survfit()” in the package “survival”. P-values for the difference between two races in patient survival time is calculated by the function *coxph()* in the package “survival”^11^ and the function *rmst2()* in the package “survRM2”^12^. In the implementations, patient-age at the initial clinical date is included as a covariate and the default arguments are used. The functions *wilcox()* and *lm()* in the package “stats” are used in the Mann Whitney test and linear regression analysis, respectively.

## Results

Among the 33 cancer types with clinically-annotated multi-omic data available at the TCGA database by April 24, 2015, sixteen are studied in this work. Each of the selected cancer types has at least 14 patients from a minority population (i.e. black or Asian Americans) besides the dominant white Americans (Table 1). The studied cancer types include bladder urothelial carcinoma (BLCA), glioblastoma multiforme (GBM), head and neck squamous cell carcinoma (HNSC), kidney renal clear cell carcinoma (KIRC), lung adenocarcinoma (LUAD), lung squamous cell carcinoma (LUSC), breast invasive carcinoma (BRCA), ovarian serous cystadenocarcinoma (OV), uterine corpus endometrial carcinoma (UCEC), colon adenocarcinoma (COAD), thyroid carcinoma (THCA), cervical squamous cell carcinoma and endocervical adenocarcinoma (CESC), esophageal carcinoma (ESCA), kidney renal papillary cell carcinoma (KIRP) liver hepatocellular carcinoma (LIHC), and stomach adenocarcinoma (STAD). The sample sizes of those cancer types range from 171 to 967.

### Racial disparity in cancer incidence rate

We use a naïve binomial test to estimate the p-value for the difference of cancer incidence rates between black (or Asian) and white groups for each cancer type (Results are presented in Supplementary Table 3 and the method is outlined in the table notes). We find that, except for Black versus White in three cancers (i.e. BRCA, CESC and ESCA) and Asian versus White in one cancer (i.e. THCA), all the other differences are significant (*p* < 0.01).

### Racial disparity in patient survival

For each TCGA cancer type, the samples not belonging to the white, black or Asian racial groups are excluded from the survival analysis. Two statistical methods are employed. One is the conventional Cox proportional hazard (Cox-PH) regression, and the other is the Restricted Mean Survival Time (RMST)^13^. Compared to a Cox-PH model, RMST has an advantage in alleviating the potential low efficiency, which may happen when the Kaplan Meier survival curves of two groups substantially deviate from parallelism and/or cover different age domains. However, its implementation needs a cut-off for survival time, potentially leading to the loss of information and statistical power. In our analysis, the significance of a group comparison is determined by an aggregated p-value (*p*), which integrates *p*_*COX*__−__*PH*_ (the p-value obtained from the Cox-PH analysis) and *p*_*RMST*_ (the p-value obtained from the RMST method) by the conventional Bonferroni method^14^. The formula is *p* = K×min(*p*_*COX−PH*’_ *p*_*RMST*_), where K = 2.

**Figure 1:**
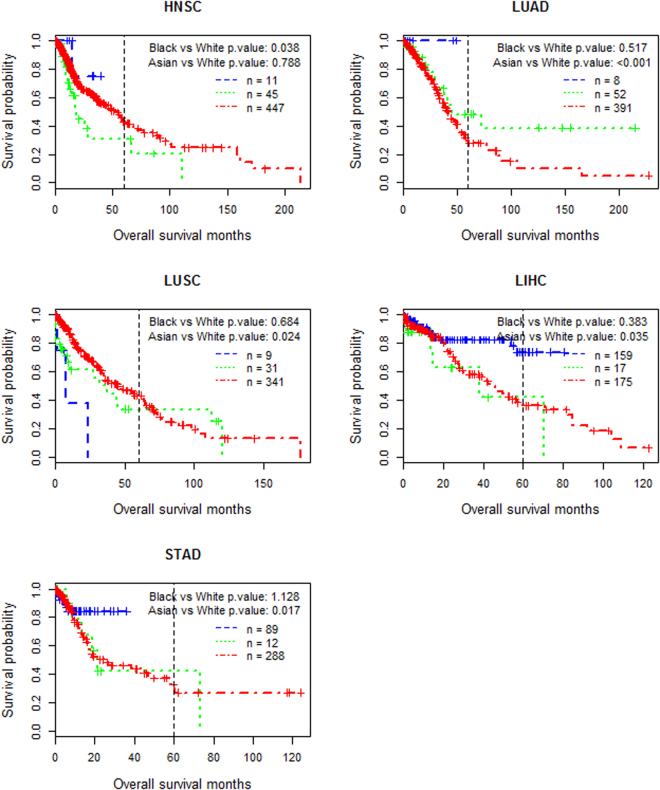
Racial disparity in survival time of cancer patients. Red: White patients; Green: Black patients; Blue: Asian patients. Censored patients (samples), for whom the follow-up after treatment ends before events (death) occur, are marked with vertical ticks. For each comparison, the printed p-value is the aggregated p-value (*p*), which integrates *p*_*COX−PH*_ (the p-value obtained from the Cox-PH analysis) and *p*_*RMST*_ (the p-value obtained from the RMST method) by the conventional Bonferroni method.

Five cancer types demonstrate racial disparities in the overall survival time of patients (Fig. 1). The first is HNSC, in which the survival in black patients is significantly worse than that in white patients (*p* = 0.038). The second is LUAD in which Asian patients show a nearly perfect survival profile. Although the Asian group contains only eight samples, the comparison with white patients is extremely significant (*p* < 0.001). Black patients demonstrate a beyond-five-year survival advantage over white patients but the difference is not significant (*p* > 0.05). Nevertheless, the difference of ten-year survival rates between the black and white patients is significant (*p*−*value* = 0.002) if Fisher’s Exact Test is used. The third is LUSC, in which none of the nine Asian patients lived more than three years and their survival is significantly poorer compared to white patients (*p* = 0.024). For the last two cancer types, i.e. LIHC and STAD, the p-values of the comparisons between Asian and white groups are 0.035 and 0.017, respectively. The Asian group also demonstrates much desired survival rates (over 80%) until 40 months. In particular, the survival advantage of Asian patients over white and black STAD patients is still substantial after 90 months from the initial clinical dates.

### Racial disparity in tumor mutation burdens

By a Mann Whitney test, in which the null hypothesis is that the mean ranks of the groups are the same, we evaluate the between-race differences of mutation burdens (i.e. the numbers of somatic mutations) in three gene sets (or catalogues). The first, pcDriver, contains 291 (high-confident) driver genes identified by a pan-cancer project^15^. The second consists of the 506 cancer genes collected by the Cancer Gene Census of COSMIC (Catalogue of Somatic Mutations in Cancer) database^16^. The third includes all the HUGO genes for which official symbols have been approved by the Human Genome Organization Nomenclature Committee. It is worth noting that, if a gene has two or multiple mutations in an individual sample, each of those mutations will be counted towards the calculation of mutation burden.

As shown in Table 2, racial disparities (*p* < 0.05) are observed in five cancer types regarding the mutations present in the pcDriver genes. Specifically, in BLCA, a median white patient has 11 driver mutations, 4 more than that of an average Asian patient. A similar but less significant pattern is found in KIRC. Among LUAD patients, black patients have heavier driver mutation burden compared to white patients. Their medians are 13 and 9, respectively. On the other hand, white patients suffer more mutations than black patients for UCEC and KIRP. In addition, the difference between black and Asian patients is significant in UCEC.

**Table 2:** The statistics of non-synonymous somatic mutations in the pan-cancer driver (pcDriver) genes^‡^.

Cancer	White			Black			Asian			P-value		
	Q1	Q2	Q3	Q1	Q2	Q3	Q1	Q2	Q3	White::Black	White::Asian	Black::Asian
BLCA	7	11	16	5	8	13	3.5	7	11	1.9E-01	**5.0E-03**	4.7E-01
GBM	2	4	5	3	4	6	2	4	7	2.0E-01	7.2E-01	9.7E-01
HNSC	4	7	11	4.75	8.5	11	3.5	6	13	5.6E-01	7.7E-01	7.2E-01
KIRC	2	3	5	1.25	3.5	4	1	2	2	3.0E-01	**1.5E-02**	2.9E-01
LUAD	5	9	15	10	13	18	5.75	9.5	18.25	**6.8E-03**	6.2E-01	4.6E-01
LUSC	6	10	14	6	10	14	4	5	10	1.0E+00	1.4E-01	1.6E-01
BRCA	1	3	4	1	3	4	2	3	5	7.9E-01	1.4E-01	1.4E-01
OV	1	2	3	2	3	3	1	2.5	4.25	2.4E-01	5.8E-01	8.9E-01
UCEC	5	8	15	4	5	12	5	7	60	**2.4E-02**	3.4E-01	**1.7E-02**
COAD	5	7	12	5.5	7	15.5	5	10	73	9.3E-01	3.5E-01	4.7E-01
THCA	1	1	2	1	1	2	1	1	1	6.7E-01	6.3E-01	5.6E-01
CESC	2	3	7	2	3.5	6.25	1	2	4.5	9.3E-01	8.1E-02	2.0E-01
ESCA	3	5	7	6.25	6.5	6.75	3	5	6	2.7E-01	8.4E-01	2.0E-01
KIRP	1	2	4	1	1	2.5	0.75	1.5	2.25	**2.7E-02**	5.0E-01	8.2E-01
LIHC	2	3.5	5	3	4	6.5	2	3	6	2.0E-01	7.9E-01	3.2E-01
STAD	3	6	10.5	4	19.5	36.25	2	4	11	3.0E-01	4.0E-01	2.2E-01

^‡^Q1, Q2 and Q3 are the first quantile, the second quantile (median) and the third quantile of mutation numbers, respectively. The number of tumor samples in each cancer-race group is the same as that in Table 1. P-values are calculated by the Mann Whitney test.

We also observe the racial disparities in BLCA, KIRC and LUAD, but not UCEC and KIRP, regarding the mutations present in the COSMIC genes and HOGO genes (Supplementary Tables 4 and 5). The analysis of these two gene catalogues also shows some racial disparities that are not detected in the analysis of the pcDriver genes. Several cancers, including BRCA, CESC, OV and ESEA, are involved.

### Relationship between tumor mutation burden and cancer incidence rate

We further investigate whether the observed mutational disparities can explain the variability of cancer incidence by a set of statistical analyses. In these analyses, we treat the combination of a racial group and a cancer type as an “experimental” unit, whose incidence and mutation quantities constitute an observation (or record) in the working dataset.

**Figure 2:**
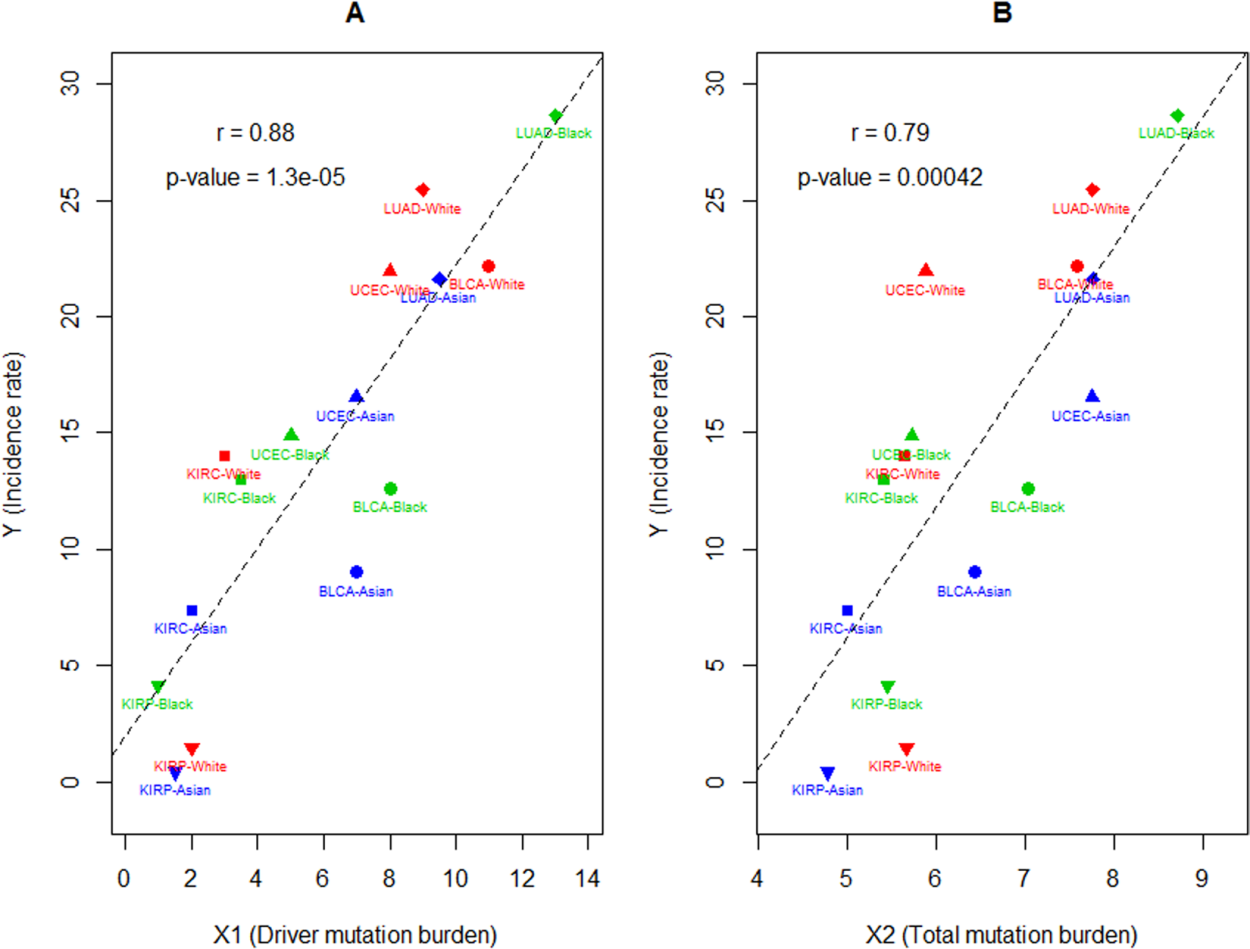
The association between mutation burden and cancer incidence rate for the five cancer types that demonstrate mutational disparities between patient races. Each data point represents the combination of a racial group and a TCGA cancer. Y (Incidence rate) in the both plots indicates the number of new cancer cases per 100000 individuals per year. (**A**) X1 indicates the median of mutation numbers in the pan-cancer driver genes. (**B**) X2 indicates the log2 transformed median of mutation numbers in all HUGO genes. The p-value of Pearson correlation (r) between X1 (X2) and Y is estimated by the t-test. The regression of Y on X1 (X2) is denoted by the dotted red line.

The first analysis (AS-1) focuses on the five cancers that demonstrate mutational disparities in driver genes (highlighted in Table 2). The association between cancer incidence rate and the number of mutations in the pan-cancer driver (pcDriver) genes or the log2 transformed number of mutations in the HOGO genes is estimated by the Pearson correlation (r). As showed in Fig. 2, the association is quite strong (*r* = 0.88 *or* 0.79, *p* < 0.00002 *or* 0.005) and the pattern approximately demonstrates a linear relationship.

**Figure 3:**
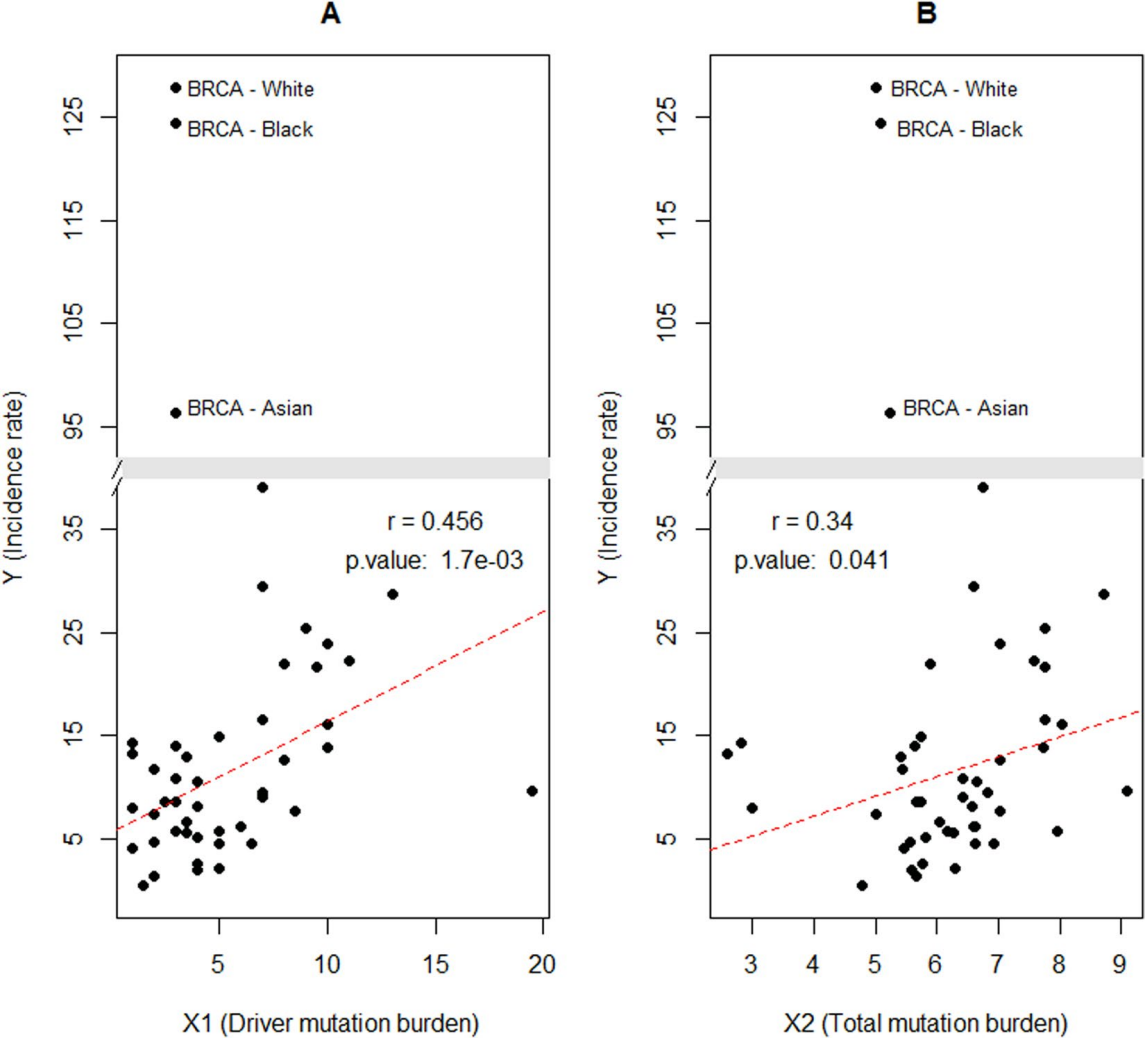
The association between mutation burden and cancer incidence rate for all the addressed cancer types except for BRCA. Y (Incidence rate) in the both plots indicates the number of new cancer cases per 100000 individuals per year. Each data point represents the combination of a racial group and a TCGA cancer. (**A**) 1 indicates the median of mutation numbers in the pan-cancer driver genes. (**B**) X2 indicates the log2 transformed median of mutation numbers in all HUGO genes. The p-value of Pearson correlation (r) between X1(X2) and Y is estimated by the t-test. The regression of Y on X1 (or X2) is denoted by the dotted red line. The information of BRCA is not used in the analysis. The graphics is generated by the *gap.plot()* function in the R package “plotrix”.

The second analysis (AS-2) repeats the correlation tests using the information of 15 cancers (of the 16 cancers listed in Table 1). BRCA is excluded from the analysis because its extremely-high incidence rates could dominate the parameter estimation. The results (Fig. 3) largely confirm the positive association between cancer incidence and mutation burden observed in AS-1.

The third analysis (AS-3) is based on the information of 8 cancers, i.e. COAD, ESCA, GBM, HNSC, LIHC, LUAD, LUSC and THCA, which are a subset of the 31 cancers studied by Tomasetti and Vogelstein^10^. The effects of mutation burden and the lifetime number of stem cell divisions (SCD, Supplementary Table 6) on cancer incidence are evaluated by five regression models (Table 3). The results show that driver mutation burden (DM) can explain ~25% of the variability of cancer incidence across cancer types and racial groups, similar to the percentage explained by cell divisions. The model containing both DM and SCD as the explanatory variables is more predictive (*R* ^2^ = 0.374) than the models with either DM or SCD as the only explanatory variable.

**Table 3:** The regression of cancer incidence on stem cell division and somatic mutation burden.

Model	Explanatory variable^a^	Adjusted-R^2^	p-value (SCD)	p-value (DM)	p-value (TSM)
Model-1	SCD, DM	0.374	0.030	0.042	NA
Model-2	SCD, TSM	0.340	0.011	NA	0.079
Model-3	SCD	0.268	0.006	NA	NA
Model-4	DM	0.247	NA	0.008	NA
Model-5	TSM	0.139	NA	NA	0.041

^a^SCD: the lifetime number of stem cell divisions. DM: the number of somatic mutations in the pan-cancer driver (pcDriver) genes. TSM: the number of somatic mutations present in all HOGO genes. Before the regression analysis, the logarithm transformation is applied to SCD and TSM.

### Further analysis on the relationship between mutation burden and cancer incidence rate

#### AS-S1

Not all non-synonymous mutations occurring in pcDriver genes are driver mutations. DNA bases in which driver mutations occur tend to be, but not necessarily are, conservative in mammalian evolution^17,18^. In this regard, the number of mutations in pcDriver genes in a tumor should be considered as an estimate (or a representative metric) of its driver mutation burden.

Of the total 33400 non-synonymous pcDriver gene mutations in all 4839 tumor samples analyzed in this study, 29623 (amounting to 88.7%) are single nucleotide variations (SNVs). The others include 11 double nucleotide polymorphisms (DNPs) and 3766 indels. We retrieve the PHRED-like deleteriousness scores (Scaled C-Scores) of these SNVs from Combined Annotation Dependent Depletion (CADD) (http://cadd.gs.washington.edu/)^19^. We find that 86.6% of the obtained scores are larger than 15 (a mutation with its C-Score over 15 is expected to be among the 3.2% of the most deleterious SNVs), the cutoff recommended by CADD for the identification of pathogenic variations. Among the 48 cancer-race groups, only KIRP-Asian group has the average Scaled C-Score (16.7) less than 20 (Supplementary Table 7).

By filtering the less deleterious (Scaled C-Score <15) SNVs from the mutation list of pcDriver genes, we generate an alternative estimate (or metric) of the driver mutation burden of a tumor sample. We find that the association pattern and strength (Supplementary Figure 1A) between this parsimoniously-measured mutation burden and cancer incidence rate are very similar to those shown Fig. 3A. This implies that the noise potentially introduced in measuring driver mutation burden do not seriously impact the validity of the findings presented in the previous subsection.

#### AS-S2

The mutations not occurring in cancer driver genes are typically known as passenger mutations. Passenger mutation burden is a proven, both empirically and theoretically, positive predictor for driver mutation burden^20^. In the TCGA data, passenger mutations amount to ~93% of the total mutations. We calculate and test the correlation between the passenger mutation burden and cancer incidence rate (Supplementary Figure 1B). The result is similar to that between the total mutation burden and cancer incidence rate (Fig. 3B).

## Discussion

In the literature, the mortality of a cancer and the variability across different racial groups are usually determined by epidemiological data^7–9,21–28^. In this paper, we perform an integrative analysis of the clinical and genomic data of the TCGA tumor samples, finding racial disparities present in five cancer types with regard to the survival profile of patients. We also notice that, although some racial disparities observed from the analysis of epidemiological data are not identified due to the relatively small sample sizes of the minor racial groups, the Kaplan Meier curves still provide insight into the nature of these disparities. For example, it is well known that black lung cancer patients have a higher death rate compared to white patients^21^ and our result implies that the disparity is mainly due to the lower short-time survival chances of black LUSC patients. This is consistent with the opinion that the treatment of black patients has been more frequently delayed due to socioeconomic factors^21,26,27,29^.

Personalized medicine is a new and exciting research field, being considered as the future of cancer patient management^30^. The potential strength depends on the understanding of the biological and genetic characteristics of individual tumors^31^, for which the differences between racial populations may be an information source. In this study, we found that the numbers of tumor driver mutations are differentiated (p < 0.05) over the racial groups in five cancers. Theoretically, both genetic and environmental factors can contribute to these disparities. However, the detailed stories should vary, depending on cancer types. For example, the mutational disparity in LUAD is indicated by the small p-value for the White::Black comparison and is characterized by the high mutation burden in black patients. Since, among people of low socioeconomic status, black Americans have a higher smoking rate than the white^32^, it could not be too bold to attribute the mutational disparity to an environment factor. On the other hand, the racial disparity in BLCA is indicated by the small p-value for the White::Asian comparison and is characterized by the high mutation burden in white patients. Because there is no evidence showing that the lifestyles and diets of the black, whose mutational profile is similar to the Asian, are closer to the Asian than the white, the observed disparity in somatic mutations may be due to a genetic factor. These speculations warrant further validation with more relevant data.

The most remarkable finding of our work is that there is a significant positive correlation between the incidence rate and the race-specific median (driver) mutation burden of a cancer. This association seems to deviate from the well-known perception that relates cancer incidence rate to the total number of (driver) mutations that can be accumulated in a tissue during the lifespan of a person. The reason is that the measurement of mutation burden in a tumor is irrelevant to the size of stem cell populations (or the divisions) that varies substantially in an exponential scale across tissues. A potential explanation for the paradox is that: the requirement for driver mutations to develop cancer in a tissue with a large population of stem cells (and/or being readily subject to mutagens) could be relatively high but the precancerous cells meeting the threshold in such a tissue still outnumber the precancerous cells in a “smaller” (and/or “safe”) tissue. Similar hypotheses have been proposed to explain the famous Peto’s paradox, i.e. biological species of larger body mass and/or longer lifespan exhibit smaller than expected incidences of cancer^33^. Different from the “bad luck” theory that attributes cancer to random mutations^10^, our results indicate the causal complexity of cancer. That is, besides tissue types, the race-related genetic and environmental factors are among the mediators for the association between the variabilities of mutation burden and disease incidence across tissues. Theoretically, mutation burden in a tumor is directly related to the number of somatic cells derived from a single stem cell. In this regard, there is a similarity between our result and that reported by Noble *et al*.^34^. The publication shows that both components of the lifetime number of stem cell divisions, i.e. standing stem cell number and per stem cell lifetime replication rate, have a statistically significant and independent effect on explaining variation in cancer incidence over the 31 cancer types studied by Tomasetti and Vogelstein^10^.

## Electronic supplementary material

Supplementary Information
