# Supplementary material for: Racial disparities in patient survival and tumor mutation burden, and the association between tumor mutation burden and cancer incidence rate
Source: Sci Rep. 2017 Oct 20;7:13639. doi: 10.1038/s41598-017-13091-y (PMC5651797; doi:10.1038/s41598-017-13091-y)
Supplement: Supplementary file 1 — Supplementary Information [file 41598_2017_13091_MOESM1_ESM.doc]

**Racial disparities in patient survival and tumor mutation burden, and the association between tumor mutation burden and cancer incidence rate**

Wensheng Zhang^1^, Andrea Edwards^1^, Erik K. Flemington^2^, Kun Zhang^1§^

^1^Department of Computer Science, Bioinformatics facility of Xavier RCMI Center of Cancer Research, Xavier University of Louisiana, 1 Drexel Drive, New Orleans LA 70125

^2^Tulane School of Medicine, Tulane Cancer Center, Tulane University, 1700 Tulane Ave, New Orleans, LA 70112

§ Corresponding author

Email addresses:

WZ: [wzhang@xula.edu](mailto:wzhang@xula.edu)

AE: aedwards@xula.edu

EKF: [eflemin@tulane.edu](mailto:eflemin@tulane.edu)

KZ: kzhang@xula.edu

| **Supplementary Table 1: Archives and file names of mutation and clinical data** |
| --- |
|  |
| **Section 1: Cancer names** |
| Bladder urothelial carcinoma [BLCA] |
| Glioblastoma multiforme [GBM] |
| Head and neck squamous cell carcinoma [HNSC] |
| kidney renal clear cell carcinoma (KIRC) |
| Lung adenocarcinoma [LUAD] |
| Lung squamous cell carcinoma [LUSC] |
| Breast invasive carcinoma [BRCA] |
| Ovarian serous cystadenocarcinoma [OV] |
| Uterine corpus endometrial carcinoma [UCEC] |
| Cervical squamous cell carcinoma and endocervical adenocarcinoma [CESC] |
| esophageal carcinoma (ESCA) |
| kidney renal papillary cell carcinoma (KIRP) |
| Colon adenocarcinoma [COAD] |
| Esophageal carcinoma [ESCA] |
| Liver hepatocellular carcinoma [LIHC] |
| Stomach adenocarcinoma [STAD] |
|  |
| **Section 2: Archive names of somatic mutation data** |
| broad.mit.edu_BLCA.IlluminaGA_DNASeq_automated.Level_2.1.4.0 |
| broad.mit.edu_GBM.IlluminaGA_DNASeq.Level_2.100.1.0 |
| broad.mit.edu_HNSC.IlluminaGA_DNASeq_automated.Level_2.1.4.0 |
| hgsc.bcm.edu_KIRC.Mixed_DNASeq.Level_2.1.2.0 |
| broad.mit.edu_LUAD.IlluminaGA_DNASeq_automated.Level_2.1.5.0 |
| broad.mit.edu_LUSC.IlluminaGA_DNASeq.Level_2.100.1.0 |
| genome.wustl.edu_BRCA.IlluminaGA_DNASeq_curated.Level_2.1.1.0 |
| genome.wustl.edu_OV.IlluminaGA_DNASeq.Level_2.2.1.0 |
| genome.wustl.edu_UCEC.IlluminaGA_DNASeq.Level_2.1.7.0 |
| ucsc.edu_CESC.IlluminaGA_DNASeq_automated.Level_2.1.1.0 |
| ucsc.edu_ESCA.IlluminaGA_DNASeq_automated.Level_2.1.0.0 |
| ucsc.edu_KIRP.IlluminaGA_DNASeq_automated.Level_2.1.2.0 |
| hgsc.bcm.edu_COAD.IlluminaGA_DNASeq.Level_2.1.5.0 |
| ucsc.edu_ESCA.IlluminaGA_DNASeq_automated.Level_2.1.0.0 |
| ucsc.edu_LIHC.IlluminaGA_DNASeq_automated.Level_2.1.1.0 |
| hgsc.bcm.edu_STAD.IlluminaGA_DNASeq_automated.Level_2.1.0.0 |
|  |
| **Section 3: File names of somatic mutation data** |
| PR_TCGA_BLCA_PAIR_Capture_All_Pairs_QCPASS_v5.aggregated.capture.tcga.uuid.automated.somatic.maf |
| step4_gbm_liftover.aggregated.capture.tcga.uuid.maf2.4.migrated.somatic.maf |
| PR_TCGA_HNSC_PAIR_Capture_All_Pairs_QCPASS_v4.aggregated.capture.tcga.uuid.automated.somatic.maf |
| hgsc.bcm.edu_KIRC.Mixed_DNASeq.1.somatic.maf |
| PR_TCGA_LUAD_PAIR_Capture_All_Pairs_QCPASS_v4.aggregated.capture.tcga.uuid.automated.somatic.maf |
| step4_LUSC_Paper_v8.aggregated.tcga.maf2.4.migrated.somatic.maf |
| genome.wustl.edu_BRCA.IlluminaGA_DNASeq.Level_2.1.1.0.curated.somatic.maf |
| mit.100.1.0_wustl.2.1.3.0_wustl.2.2.0.0.maf |
| genome.wustl.edu_UCEC.IlluminaGA_DNASeq.Level_2.1.7.somatic.maf |
| genome.wustl.edu_UCEC.IlluminaGA_DNASeq.Level_2.1.7.somatic.maf |
| ucsc.edu_ESCA.IlluminaGA_DNASeq_automated.Level_2.1.0.0.somatic.maf |
| ucsc.edu_KIRP.IlluminaGA_DNASeq_automated.Level_2.1.2.0.somatic.maf |
| hgsc.bcm.edu_COAD.IlluminaGA_DNASeq.1.somatic.maf |
| ucsc.edu_ESCA.IlluminaGA_DNASeq_automated.Level_2.1.0.0.somatic.maf |
| ucsc.edu_LIHC.IlluminaGA_DNASeq_automated.Level_2.1.1.0.somatic.maf |
| hgsc.bcm.edu_STAD.IlluminaGA_DNASeq.1.somatic.maf |
|  |
| **Section 4: Archive names of clinical data** |
| nationwidechildrens.org_BLCA.bio.Level_2.0.27.0 |
| nationwidechildrens.org_GBM.bio.Level_2.0.25.0 |
| nationwidechildrens.org_HNSC.bio.Level_2.0.21.0 |
| nationwidechildrens.org_KIRC.bio.Level_2.0.21.0 |
| nationwidechildrens.org_LUAD.bio.Level_2.0.24.0 |
| nationwidechildrens.org_LUSC.bio.Level_2.0.21.0 |
| nationwidechildrens.org_BRCA.bio.Level_2.0.24.0 |
| nationwidechildrens.org_OV.bio.Level_2.0.23.0 |
| nationwidechildrens.org_UCEC.bio.Level_2.0.22.0 |
| nationwidechildrens.org_CESC.bio.Level_2.0.36.0 |
| nationwidechildrens.org_ESCA.bio.Level_2.0.34.0 |
| nationwidechildrens.org_KIRP.bio.Level_2.0.34.0 |
| nationwidechildrens.org_COAD.bio.Level_2.0.23.0 |
| nationwidechildrens.org_ESCA.bio.Level_2.0.34.0 |
| nationwidechildrens.org_LIHC.bio.Level_2.0.38.0 |
| nationwidechildrens.org_STAD.bio.Level_2.0.31.0 |
|  |
| **Section 5: File names of clinical data** |
| nationwidechildrens.org_clinical_patient_blca.txt |
| nationwidechildrens.org_clinical_patient_gbm.txt |
| nationwidechildrens.org_clinical_patient_hnsc.txt |
| nationwidechildrens.org_clinical_patient_kirc.txt |
| nationwidechildrens.org_clinical_patient_luad.txt |
| nationwidechildrens.org_clinical_patient_lusc.txt |
| nationwidechildrens.org_clinical_patient_brca.txt |
| nationwidechildrens.org_clinical_patient_ov.txt |
| nationwidechildrens.org_clinical_patient_ucec.txt |
| nationwidechildrens.org_clinical_patient_cesc.txt |
| nationwidechildrens.org_clinical_patient_esca.txt |
| nationwidechildrens.org_clinical_patient_kirp.txt |
| nationwidechildrens.org_clinical_patient_coad.txt |
| nationwidechildrens.org_clinical_patient_esca.txt |
| nationwidechildrens.org_clinical_patient_lihc.txt |
| nationwidechildrens.org_clinical_patient_stad.txt |

**Supplementary Table 2** The alignment of the TCGA cancers and SEER (Surveillance, Epidemiology, and End Results Program) cancers and incidence rate adaptation

|  | Incidence rate (new cases per 10,0000 individuals per year ) | | |  |  | |  | |
| --- | --- | --- | --- | --- | --- | --- | --- | --- |
| TCGA cancer type | White | Black | Asian | SEER cancer type | | Weighted† | | Note‡ |
| BLCA | 22.2 | 12.6 | 9 | Urinary Bladder (Invasive and In Situ) | | no | |  |
| GBM | 5.2 | 2.51 | 1.94 | Glioblastoma ^1^ | | --- | |  |
| HNSC | 9.47 | 7.64 | 6.26 | Oral Cavity and Pharynx (Invasive) | | yes, generally | | Based on SEER |
| KIRC | 14 | 13 | 7.38 | Kidney And Renal Pelvis (Invasive) | | yes, race-specifically | | Based on Olshan et al (2013) ^2^ |
| LUAD | 25.47 | 28.68 | 21.61 | Lung and Bronchus (Invasive) | | yes, race-specifically | | Based on SEER |
| LUSC | 13.79 | 16.13 | 5.75 | Lung and Bronchus (Invasive) | | yes, race-specifically | | Based on SEER |
| BRCA | 127.9 | 124.4 | 96.3 | Breast (Invasive) | | no | |  |
| OV | 11.8 | 8.6 | 8.52 | Ovary (Invasive) | | yes, race-specifically | | Based on SEER |
| UCEC | 21.96 | 14.88 | 16.52 | Corpus and Uterus, NOS (Invasive) | | yes, race-specifically | | Based on Wright, et al (2009) ^3^ |
| COAD | 29.5 | 39.1 | 23.9 | Colon (Invasive) | | no | |  |
| THCA | 14.3 | 8 | 13.2 | Thyroid (Invasive) | | no | |  |
| CESC | 5.7 | 6.58 | 4.68 | Cervix Uteri (Invasive) | | yes, race-specifically | | Based on SEER |
| ESCA | 4.6 | 4.6 | 2.2 | Esophagus (Invasive) | | no | |  |
| KIRP | 1.45 | 4.16 | 0.41 | Kidney And Renal Pelvis (Invasive) | | yes, race-specifically | | Based on Olshan et al (2013) ^2^ |
| LIHC | 5.55 | 8.1 | 10.78 | Liver and Intrahepatic Bile Duct (Invasive) | | yes, race-specifically | | Based on SEER |
| STAD | 6.16 | 9.64 | 10.53 | Stomach (Invasive) | | yes, race-specifically | | Based on SEER |

^†^ “no” indicates that the incidence rates of the TCGA cancer (Cancer-A) are estimated by the rates of the SEER cancer (Cancer-B); “yes, generally” indicates that the incidence rates of Cancer-A are estimated by the products of the rates of Cancer-B and a weight that represents the proportion of the tumor cases of Cancer-A among the total cases of Cancer-B. “yes, race-specifically” indicates that the incidence rates of Cancer-A are estimated by the products of the rates of Cancer-B and three race-specific weights that represent the proportions of the tumor cases of Cancer-A among the total cases of Cancer-B.

^‡^ Indicates information source of the distribution of the historical subtypes of the SEER cancer, on which the adaptation of incidence rates from the SEER cancer to the TCGA cancer is based.

**Supplementary Table 3** Racial disparity in cancer incidence rate (new cases per 100000 individuals per year)

| Cancer | Difference  (Black-White) | p-value ‡  (Black-White) | Diff  (Asian-White) | p-value  (Asian-White) |
| --- | --- | --- | --- | --- |
| BLCA | -9.6 | 1.00E-108 | -13.2 | 7.00E-165 |
| GBM | -2.69 | 5.00E-39 | -3.26 | 3.00E-45 |
| HNSC | -1.83 | 8.00E-10 | -3.21 | 1.00E-21 |
| KIRC | -1 | 8.00E-03 | -6.62 | 1.00E-62 |
| LUAD | 3.21 | 5.00E-10 | -3.86 | 2.00E-11 |
| LUSC | 2.34 | 8.00E-10 | -8.04 | 1.00E-98 |
| BRCA | -3.5 | 3.00E-02 | -31.6 | 1.00E-70 |
| OV | -3.2 | 7.00E-12 | -3.28 | 1.00E-09 |
| UCEC | -7.08 | 1.00E-29 | -5.44 | 2.00E-13 |
| COAD | 9.6 | 2.00E-63 | -5.6 | 6.00E-20 |
| THCA | -6.3 | 2.00E-73 | -1.1 | 1.00E-02 |
| CESC | 0.88 | 1.00E-02 | -1.02 | 9.00E-03 |
| ESCA | 0 | 1.00E+00 | -2.4 | 2.00E-26 |
| KIRP | 2.71 | 6.00E-75 | -1.04 | 2.00E-18 |
| LIHC | 2.55 | 5.00E-24 | 5.23 | 6.00E-64 |
| STAD | 3.48 | 3.00E-38 | 4.37 | 4.00E-43 |

‡ The two-tail p-value is calculated using the function *binom.test (x, n, pw)* in the R package “stats”. For a cancer occurs to both men and women, *n* is the total number of black SEER participants. For a cancer specific to females, *n* is half of the total number of black SEER participants. *x* represents the registered new cancer cases from black participants. It is estimated by
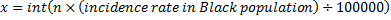
, in which *int(.)* denotes the function for coercing a numeric value to the nearest integer. *pw* is the probability that cancer occurs in a white SEER participant per year, estimated by
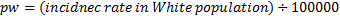
. The null hypothesis of the test is that black population has the same cancer incidence rate as white population whose participants are predominant in SEER program. The basic assumption of the test is that the SEER’s measure of cancer incidence rate for white population is sufficiently accurate. The p-value for the Asian-White comparison is calculated using the same method. The sizes of SEER participants in 2010 are 9975844 and 7390717 (used in this analysis) for black and Asian, respectively (<https://seer.cancer.gov/registries/data.html>).

**Supplementary Table 4** Statistics of non-synonymous somatic mutations in the COSMIC census cancer genes^‡^

|  | White | | | Black | | | Asian | | | P-value | | |
| --- | --- | --- | --- | --- | --- | --- | --- | --- | --- | --- | --- | --- |
| Cancer | Q1 | Q2 | Q3 | Q1 | Q2 | Q3 | Q1 | Q2 | Q3 | White::Black | White::Asian | Black::Asian |
| BLCA | 6 | 11 | 16 | 5 | 10 | 17 | 3.25 | 6 | 8.75 | 5.4E-01 | **8.6E-04** | 1.7E-01 |
| GBM | 2 | 3 | 5 | 2 | 4 | 5 | 1 | 4 | 7 | 5.7E-01 | 5.9E-01 | 6.3E-01 |
| HNSC | 4 | 6 | 10 | 4 | 7 | 10 | 3.5 | 5 | 8.5 | 7.4E-01 | 4.0E-01 | 3.8E-01 |
| KIRC | 2 | 3 | 5 | 0.25 | 2 | 4.5 | 0.5 | 1 | 2.5 | **3.3E-02** | **1.0E-02** | 5.7E-01 |
| LUAD | 4 | 8 | 16 | 7 | 13 | 18 | 5.75 | 8.5 | 16.8 | **1.3E-02** | 9.2E-01 | 2.9E-01 |
| LUSC | 6 | 9 | 13 | 7 | 11 | 12 | 7 | 8 | 10 | 6.1E-01 | 8.2E-01 | 5.5E-01 |
| BRCA | 1 | 2 | 4 | 1 | 2 | 4 | 2 | 3 | 4 | 8.5E-01 | 1.2E-02 | **3.6E-02** |
| OV | 1 | 2 | 3 | 2 | 3 | 3 | 2 | 2.5 | 4 | 1.2E-01 | 3.8E-01 | 8.2E-01 |
| UCEC | 5 | 7 | 13 | 3 | 6 | 9 | 4 | 9 | 46 | 9.8E-02 | 3.3E-01 | 1.4E-01 |
| COAD | 5 | 7 | 10 | 5 | 7 | 10 | 5.5 | 6 | 65 | 9.2E-01 | 4.2E-01 | 4.5E-01 |
| THCA | 1 | 1 | 2 | 0.25 | 1 | 2 | 1 | 1 | 2 | 9.0E-01 | 9.3E-01 | 8.8E-01 |
| CESC | 1 | 3 | 6 | 1 | 2 | 4.25 | 0.5 | 1 | 4 | 5.1E-01 | **2.0E-02** | 1.8E-01 |
| ESCA | 2 | 4 | 6 | 3 | 4 | 5 | 3 | 4 | 6 | 8.3E-01 | 7.1E-01 | 8.2E-01 |
| KIRP | 1 | 2 | 4.25 | 1 | 2 | 3 | 0.5 | 1 | 1.5 | 3.2E-01 | 2.7E-01 | 3.5E-01 |
| LIHC | 2 | 3 | 5 | 3.25 | 4 | 5.75 | 2 | 4 | 5 | 1.2E-01 | 2.1E-01 | 4.7E-01 |
| STAD | 3 | 5 | 9 | 5.5 | 27.5 | 50.8 | 2 | 4 | 11.3 | 1.1E-01 | 6.0E-01 | 9.4E-02 |

^‡^ Q1, Q2 and Q3 are the first quantile, the second quantile (median) and the third quantile of mutation numbers, respectively. The number of tumor samples in each cancer-race group is the same as that in Table 1. P-values are calculated by the Mann Whitney test.

**Supplementary Table 5** Statistics of non-synonymous somatic mutations in the HUGO genes^‡^

|  | White | | | Black | | | Asian | | | P-value | | |
| --- | --- | --- | --- | --- | --- | --- | --- | --- | --- | --- | --- | --- |
| Cancer | Q1 | Q2 | Q3 | Q1 | Q2 | Q3 | Q1 | Q2 | Q3 | White::Black | White::Asian | Black::Asian |
| BLCA | 122 | 191 | 306 | 88 | 131 | 289 | 51 | 86 | 186 | 1.40E-01 | **1.80E-04** | 9.30E-02 |
| GBM | 45 | 56 | 67 | 38 | 54 | 64 | 47 | 48 | 54 | 4.20E-01 | 3.10E-01 | 7.80E-01 |
| HNSC | 73 | 113 | 189 | 97 | 131 | 200 | 55 | 96 | 229 | 2.70E-01 | 4.40E-01 | 2.90E-01 |
| KIRC | 37 | 50 | 65 | 36 | 43 | 54 | 31 | 32 | 41 | 2.70E-01 | **2.10E-02** | 2.30E-01 |
| LUAD | 86 | 215 | 418 | 229 | 419 | 555 | 63 | 217 | 495 | **5.80E-03** | 9.50E-01 | 3.40E-01 |
| LUSC | 164 | 214 | 301 | 166 | 260 | 333 | 131 | 247 | 259 | 5.80E-01 | 6.70E-01 | 3.00E-01 |
| BRCA | 19 | 32 | 57 | 24 | 34 | 68 | 25 | 38 | 68 | **4.50E-02** | 1.30E-01 | 8.80E-01 |
| OV | 28 | 43 | 62 | 46 | 53 | 64 | 32 | 51 | 64 | **4.80E-02** | 4.50E-01 | 5.90E-01 |
| UCEC | 42 | 59 | 277 | 40 | 53 | 180 | 41 | 216 | 1268 | 5.40E-01 | 5.50E-01 | 4.00E-01 |
| COAD | 76 | 97 | 180 | 77 | 108 | 273 | 69 | 131 | 1861 | 7.40E-01 | 4.20E-01 | 5.70E-01 |
| THCA | 5 | 7 | 11 | 4 | 8 | 14 | 5 | 6 | 10 | 6.40E-01 | 1.60E-01 | 2.70E-01 |
| CESC | 42 | 72 | 129 | 29 | 66 | 108 | 37 | 47 | 75 | 5.40E-01 | 5.10E-02 | 5.50E-01 |
| ESCA | 71 | 99 | 139 | 101 | 123 | 144 | 59 | 79 | 102 | 7.10E-01 | **1.60E-02** | 2.70E-01 |
| KIRP | 32 | 51 | 89 | 27 | 44 | 58 | 25 | 28 | 30 | 9.90E-02 | 1.40E-01 | 2.70E-01 |
| LIHC | 52 | 78 | 105 | 73 | 96 | 122 | 42 | 86 | 112 | 1.10E-01 | 8.60E-01 | 2.20E-01 |
| STAD | 55 | 99 | 218 | 64 | 543 | 1055 | 50 | 100 | 223 | 3.80E-01 | 8.00E-01 | 3.20E-01 |

^‡^ Q1, Q2 and Q3 are the first quantile, the second quantile (median) and the third quantile of mutation numbers, respectively. The number of tumor samples in each cancer-race group is the same as that in Table 1. P-values are calculated by the Mann Whitney test.

**Supplementary Table 6 The lifetime number of stem cell divisions (SCD)**

| Cancer | SCD^†^ |
| --- | --- |
| COAD | 1.075×10^12^ |
| ESCA | 1.203×10^9^ |
| GBM | 2.700×10^8^ |
| HNSC | 3.186×10^10^ |
| LIHC | 2.709×10^11^ |
| LUAD | 9.272×10^9^ |
| LUSC | 9.272×10^9^ |
| OV | 3.428×10^7^ |
| THCA | 5.850×10^8^ |

**†** The data were retrieved from Tomasetti and Vogelstein (2015)^4^

**Supplementary Table 7** PHRED-like Scaled C-scores of non-synonymous somatic mutations in the pan-cancer driver (pcDriver) genes^‡^

|  | White | Black | Asian |
| --- | --- | --- | --- |
| BLCA | 26 | 25.7 | 25.4 |
| BRCA | 25.5 | 26.4 | 24.9 |
| CESC | 25.3 | 26 | 25 |
| COAD | 25.6 | 26.8 | 25.5 |
| ESCA | 26 | 22.8 | 26.1 |
| GBM | 25.6 | 24.3 | 28.6 |
| HNSC | 25.5 | 24.4 | 27.1 |
| KIRC | 25.8 | 23.4 | 26 |
| KIRP | 25.1 | 21.7 | 16.7 |
| LIHC | 23.3 | 24.7 | 22.9 |
| LUAD | 24.9 | 24.9 | 25.2 |
| LUSC | 25.3 | 25.7 | 25 |
| OV | 25 | 25.5 | 27.1 |
| STAD | 25.2 | 25.8 | 25.1 |
| THCA | 29.1 | 25.7 | 27.8 |
| UCEC | 26.3 | 25.7 | 26.2 |

^‡^ The number in each cell is the average of the PHRED-like scaled deleteriousness scores (Scaled C-Score) of the single nucleotide variations (SNVs) identified in all tumor samples of the corresponding cancer-race group. If a SNV occurs in two or multiple tumors, its C-Score will be used two (or multiple) times in calculating the average. The Scaled C-scores are retrieved from Combined Annotation Dependent Depletion (CADD) (<http://cadd.gs.washington.edu/>)^5^.


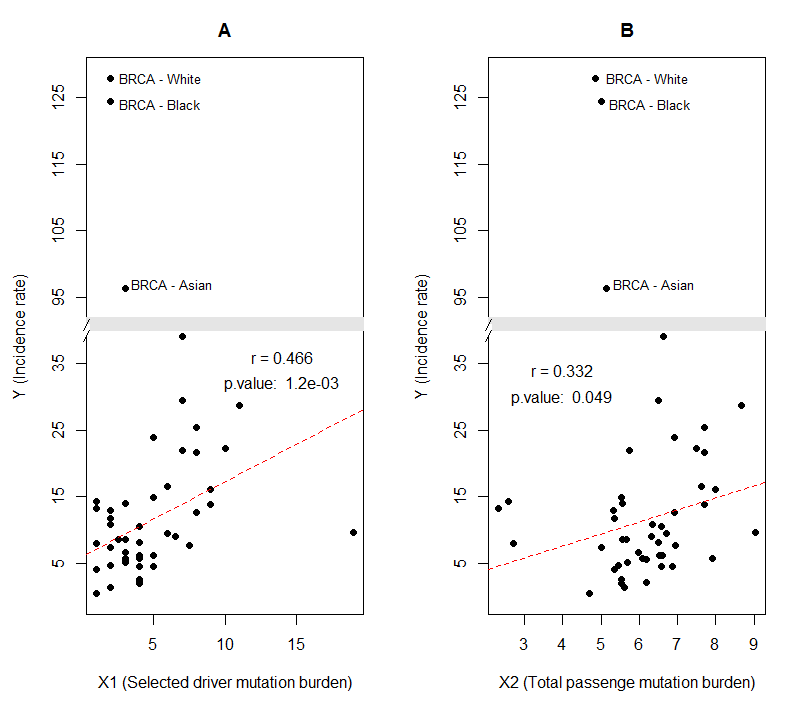


**Supplementary Figure 1** **The association between mutation burden and cancer incidence rate for all the addressed cancer types except for BRCA.** Y (Incidence rate) in both plots indicates the number of new cancer cases per 100000 individuals per year. Each data point represents the combination of a racial group and a TCGA cancer. **A**: X1 indicates the median of the numbers of the *selected (with C-score > 15 as the criterion)* mutations in the pan-cancer driver (pcDriver) genes. **B**: X2 indicates the log2 transformed median of mutation numbers in all HUGO genes *with those in pan-cancer driver genes being excluded.* The p-value of Pearson correlation (r) between X1(X2) and Y is estimated by the t-test**.** The regression of Y on X1 (X2) is denoted by the dotted red line. The *gap.plot()* function in the R package “plotrix” is used to generate the graphics.

**References:**

1 Dubrow, R. & Darefsky, A. S. Demographic variation in incidence of adult glioma by subtype, United States, 1992-2007. *BMC Cancer* **11**, 325, doi:10.1186/1471-2407-11-325 (2011).

2 Olshan, A. F. *et al.* Racial difference in histologic subtype of renal cell carcinoma. *Cancer Med* **2**, 744-749, doi:10.1002/cam4.110 (2013).

3 Wright, J. D. *et al.* Racial disparities for uterine corpus tumors: changes in clinical characteristics and treatment over time. *Cancer* **115**, 1276-1285, doi:10.1002/cncr.24160 (2009).

4 Tomasetti, C. & Vogelstein, B. Cancer etiology. Variation in cancer risk among tissues can be explained by the number of stem cell divisions. *Science* **347**, 78-81, doi:10.1126/science.1260825 (2015).

5 Kircher, M. *et al.* A general framework for estimating the relative pathogenicity of human genetic variants. *Nat Genet* **46**, 310-315, doi:10.1038/ng.2892 (2014).
